# Minimising Unnecessary Mastectomies in a Predominantly Chinese Community

**DOI:** 10.1155/2015/684021

**Published:** 2015-01-26

**Authors:** Mona P. Tan, Nadya Y. Sitoh, Yih Y. Sitoh

**Affiliations:** ^1^Breast Surgical Oncology, MammoCare, 38 Irrawaddy No. 06-21, Singapore 329563; ^2^MammoCare, 38 Irrawaddy No. 06-21, Singapore 329563; ^3^Medical Education, Mount Elizabeth Hospital, 3 Mount Elizabeth No. 17-16, Singapore 228510

## Abstract

*Background*. Recent data shows that the use of breast conservation treatment (BCT) for breast cancer may result in superior outcomes when compared with mastectomy. However, reported rates of BCT in predominantly Chinese populations are significantly lower than those reported in Western countries. Low BCT rates may now be a concern as they may translate into suboptimal outcomes. A study was undertaken to evaluate BCT rates in a cohort of predominantly Chinese women. *Methods*. All patients who underwent surgery on the breast at the authors' healthcare facility between October 2008 and December 2011 were included in the study and outcomes of treatment were evaluated. *Results*. A total of 171 patients were analysed. Two-thirds of the patients were of Chinese ethnicity. One hundred and fifty-six (85.9%) underwent BCT. Ninety-eight of 114 Chinese women (86%) underwent BCT. There was no difference in the proportion of women undergoing BCT based on ethnicity. After a median of 49 months of follow-up, three patients (1.8%) had local recurrence and 5 patients (2.9%) suffered distant metastasis. Four patients (2.3%) have died from their disease. *Conclusion*. BCT rates exceeding 80% in a predominantly Chinese population are possible with acceptable local and distant control rates, thereby minimising unnecessary mastectomies.

## 1. Introduction

As a result of prospective randomised controlled trials (RCTs) beginning in the 1970s, breast conservation therapy (BCT) was adopted as an appropriate alternative for the treatment of breast cancer and has been an accepted option for more than three decades [1–5]. A consensus statement in 1991 endorsed BCT as the surgical therapy of choice, for it offered similar survival rates while preserving the form of the breast [4]. Recent data suggests that in the presence of modern adjuvant therapies, instead of equivalent survival outcomes, BCT could be superior to mastectomy for the treatment of breast cancer [6–10]. A large retrospective analysis evaluating women with early breast cancer similar to those in one RCT demonstrated a higher 10-year breast-cancer-specific survival for women who had undergone BCT when compared with mastectomy with or without radiation [2, 6]. For patients who had characteristics unlike those in the RCTs, higher mastectomy rates were found to be associated with poorer survival outcomes [7–9], and, in a prospective series studying hormone-positive tumours, BCT resulted in lower local recurrence rates and improved survival [10].

Despite the longstanding acceptance of BCT, its utilisation in predominantly Chinese communities has been reported to be lower than in Western populations [6, 11–18]. Approximately 75–85% of women with early stage breast cancer are expected to be candidates for BCT [19], yet BCT rates in predominantly Chinese populations are reported to average 30%, even for T1-T2 tumours [11–15] (Table 1). An absolute improvement of 4% in breast-cancer-specific survival rates was reported with BCT rates of 70% [6]. It was also estimated that, for each 1-percentage-point rise in the mastectomy rate, there would be a concomitant fall in 7-year survival by 0.1% [7]. It can be inferred from these calculations that, on a population basis, no survival benefit is expected with a mastectomy rate of 70%. There is therefore a pressing need to relook at surgical treatment in predominantly Chinese communities as a persistently low BCT rate could translate to suboptimal outcomes. The reasons cited for low BCT rates include cultural preferences, surgeon bias, and factors relating to physical attributes [11–15]. Chinese women have been shown to have smaller breast tissue volume [20], which poses challenges for good cosmetic outcomes in BCT [21]. This study was therefore performed to review the authors' experience in treating women with BCT in a predominantly Chinese community, evaluate BCT rates in this cohort, and compare it with prior reports.

## 2. Materials and Methods

A retrospective analysis of all patients with breast malignancies who underwent operative treatment by clinicians at this medical facility between October 2008 and December 2011 was performed. Preoperative diagnostic workup consisted of clinical examination and standard imaging with mammography and sonography, and percutaneous needle biopsies were done for diagnosis where possible. In certain clinical settings where percutaneous biopsy was not possible or inconclusive, like where there was insufficient compression thickness or discordant imaging and biopsy results, a surgical diagnostic procedure was performed. Routine magnetic resonance imaging (MRI) was not done.

Following diagnosis, patients deemed eligible for BCT were given the option of an attempt at breast conservation or to proceed directly to mastectomy, with or without reconstruction. Eligibility for BCT was made based on the surgeon's assessment of the ability to achieve a reasonable cosmetic result after tumour resection with clear margins. Multifocal and multicentric breast cancers (MFMCBC) were not considered to be ineligible if preoperative evaluation indicated the possibility of en bloc excision of all foci through a single incision. If the tumour(s) was assessed to be too large, the patient was offered neoadjuvant medical therapy and had placement of radioopaque clip(s) prior to its commencement. These were localised before wide excision, which was guided by the position(s) of the marker clip(s).

Patients who were assessed to be eligible for BCT and who agreed to undergo a trial of BCT had wide excision of their lesion(s) performed through a single incision. Incisions were planned such that they coursed over the lesions where possible. In the instances where there were more than one tumour foci, the incision was sited above at least one of the lesions. If the other lesion(s) were more than 2 cm away from the incision, radioopaque clips were positioned in the tumour bed for ease of radiotherapy administration. Following tumour extirpation with negative margins, partial mastectomy defects were repaired using local tissue rearrangement techniques only. This was performed by mobilising full thickness parenchymal flaps off the pectoralis fascia, advancing the pillars and directly apposing them with sutures. None of the patients had volume replacement using autologous flaps or implants.

All patients who were intended for BCT had intraoperative frozen section analysis (IFSA) for margins status. If margins were positive at the time of surgery, further margins were excised until proven to be negative. These were reassessed at paraffin sections. Successful BCT of MFMCBC was defined as operative attainment of clear margins (no ink on tumour) [22] and a reasonable cosmetic outcome. In clinical scenarios where this was thought to be unattainable, mastectomy was recommended. Mastectomy was also performed for patients according to preference for breast removal rather than BCT.

The patients were referred both to a medical oncologist and to radiation oncologists for discussions relating to the need for further adjuvant treatment. Patients were considered to have completed therapy if they adhered to recommended treatment regimens. Systemic therapy was given based on the discretion of the treating medical oncologist. Whole breast irradiation was given for women who underwent BCT with a boost to the tumour bed according to the preference of the radiation oncologist. Patients who underwent mastectomy with large tumours, more than 3 positive axillary lymph nodes, and lymphovascular invasion were treated with postmastectomy radiotherapy.

Statistical analyses of the respective associations were performed using SPSS (Chicago, IL) version 11 advanced statistical software module. Comparisons of categorical variables were performed using the chi-squared test. Continuous variables with median or mean values were compared using the Student's *t*-test or Mann-Whitney *U* test where appropriate.

## 3. Results

A total of 177 female patients were treated during the study period. However, 6 were lost to follow-up before any cancer-related events were noted, leaving 171 for analysis. Clinicopathological characteristics of the cohort are summarised in Table 2. Of note, 147 patients (86%) underwent BCT. Twenty-four patients had a mastectomy. Fifteen of these (62.5%) had contraindications to BCT based on size and improbability of attaining negative margins without compromising cosmesis, while nine elected to have a mastectomy despite being eligible for BCT. The mean age of these patients who chose a mastectomy was 58 years, which was significantly higher than that of those who underwent BCT, whose mean age was 47.9 years (*P* = 0.003). There was no significant difference between the mean ages of patients who had BCT and those who underwent mastectomy out of necessity (mean age: 50.1 years) based on therapeutic principles (*P* = 0.40). The majority of the cohort was Chinese (66.7%) and was generally expected to have smaller volume breast tissue than women of other ethnicities [15, 20]. There were a fair proportion of Caucasian women and women of other ethnic origins as well, and no difference in the proportion of women undergoing BCT based on ethnicity was demonstrated (*P* = 0.88).

One hundred and fifty-six of the total of 171 (91.2%) were assessed to be suitable candidates for BCT. However, nine patients of 156 (5.8%) decided against BCT. The main reasons given by these few patients were a perceived superiority of survival with mastectomy and reduction of anxiety relating to follow-up. The mean tumour size for patients who underwent BCT was 19.2 mm, while that for women who elected for mastectomy despite being suitable candidates for BCT was 18.3 mm. There was no significant difference between the two groups (*P* = 0.83). In contrast, there was a significant difference in the mean tumour sizes of women who had BCT and those who were advised to undergo a mastectomy, the latter group of which had a mean tumour size of 40.5 mm (*P* < 0.001).

Four patients in the cohort (2.3%) required a second therapeutic surgical procedure, three for undetected multicentric tumours seen on postoperative imaging and a fourth for a falsely negative sentinel lymph node (SLN) at IFSA. The last patient had multifocal disease with two separate foci of invasive ductal carcinoma, grade III, 35 mm and 8 mm, and 2 of 22 lymph nodes involved. All four patients who needed reoperation had multifocal or multicentric breast cancers (MFMCBC) and are currently disease-free. The mean pathologic tumour size for those requiring reoperation was 32.3 mm, while those who had a single operation had a mean tumour size of 20.8 mm (*P* = 0.14). There were no patients who required a reexcision on the basis of false negative margins at IFSA.

The median follow-up period was 49 months (range 21–68 months). None of the 22 patients who presented with ductal carcinoma in situ only developed locoregional recurrence or distant metastasis. Of the other 149 patients with invasive carcinoma, two patients who underwent BCT developed local failure (1.4%). A total of four patients with invasive disease, two who had undergone BCT and two mastectomy, developed distant disease and have succumbed to their disease (2.3%). A summary of locoregional and distant events is given in Table 3.

## 4. Discussion

The last two decades of the twentieth century witnessed a paradigm shift of treatment concepts for breast cancer, with definitive evidence from prospective randomised trials demonstrating that performing less surgery resulted in equivalent survival outcomes. This led to the establishment of BCT and a steady increase in its utilisation. Unexpectedly, in the last decade, a rising trend of mastectomy rates has been observed in some Western communities [16, 23]. Despite this rise, mastectomy rates for early breast cancer in these countries are still lower than the high rates reported in predominantly Chinese populations. Mastectomy rates need to be reexamined in the presence of contemporary reports indicating possible higher cancer-specific survival, lower surgical complication rates, cost-effectiveness of treatment, and improved quality of life outcomes with BCT [6–10, 21, 24–28]. This is juxtaposed against the possible psychological benefit that mastectomy and contralateral prophylactic mastectomy (CPM) offer [24, 29], but the diminution of anxiety might not completely compensate for poorer quality of life outcomes with longer breast cancer survivorship [26, 30].

Specifically for predominantly Chinese populations, reasons cited for low BCT rates include cultural preferences, surgeon bias, and factors relating to physical attributes [11–15]. Addressing the issue of physical attributes first, Chinese women tend to have smaller volume breast (SVB) tissue which may pose a barrier to BCT [15, 20]. In this series, overall BCT rate was 85.9%. Among Chinese women, who formed two-thirds of the cohort, BCT rate was 86%. There was no significant difference of the proportion of Chinese women undergoing BCT compared with women of other ethnic groups. In particular, 80% of Caucasian women had BCT. This rate is similar to other reported series in a Western context (Table 1). This data appears to suggest that the physical attributes of Chinese women may not have a significant impact on BCT rates. In a study by Collins et al., of 125 women who were eligible for either BCT or mastectomy, 35% elected to have a mastectomy [31]. In the present study, of 156 patients who were considered eligible for BCT or mastectomy, only 5.8% decided against breast conservation. This data suggests that the local Chinese culture may be a factor in favour of BCT, rather than a condition in support of high mastectomy rates.

Another factor influencing patient decision for mastectomy is surgeon's advice [32]. In this study, 156 of 171 patients (91.2%) were assessed preoperatively to be suitable candidates for BCT. Nine patients decided in favour of mastectomy, making utilisation of BCT in this cohort 94.2%. Eligible patients who decided for mastectomy had a mean tumour size not significantly different from those who underwent successful BCT. Hence, it is reasonable to conclude that successful BCT would have been possible for this group of women, suggesting that, on the whole, eligibility of Chinese women for BCT is not significantly different from women of other ethnic origins. The difference perhaps is surgeon philosophy, which may account for varying reported mastectomy rates in different geographical locations [23, 33]. Surgeons with a strong bias toward mastectomy will require clinical circumstances extremely favourable for BCT before contemplating it, while surgeons whose default position is conservative surgery would be more inclined to explore innovative methods of achieving BCT even in challenging clinical situations. The development of oncoplastic breast surgery was likely the result of this creative pressure. There are several categories of techniques that fall into this broad description. The authors prefer the use of only volume displacement or local tissue rearrangement techniques with direct apposition of adequately mobilised residual uninvolved parenchymal pillars with sutures. This approach results in better patient satisfaction than mastopexy and avoids the issues with surgical clip migration with the use of mammoplasty techniques which require extensive tissue mobilization [21, 34, 35]. Moreover, local tissue rearrangement techniques result in lower complication rates and superior cosmetic outcomes compared to complex reconstructive techniques [21].

**Figure 1:**
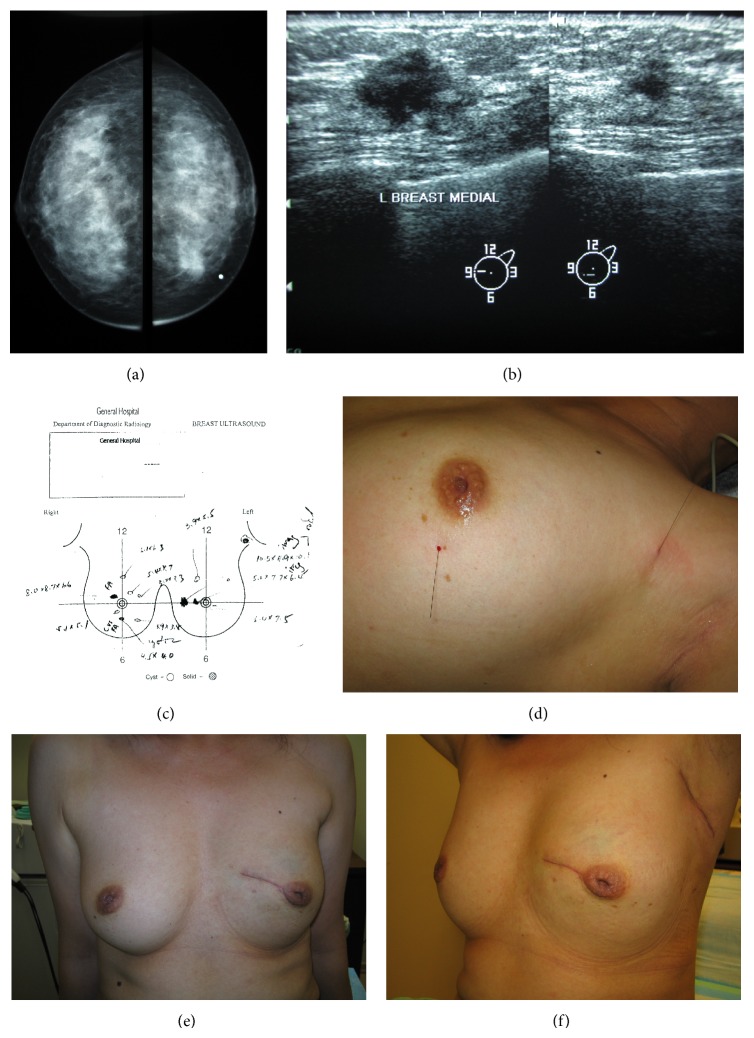
((a)–(f)) Avoiding mastectomy in a patient with “multifocal tumour” on imaging. This 45-year-old patient was diagnosed with what was thought to be multifocal invasive ductal carcinoma at another facility following core biopsy. Mastectomy was originally recommended at the first centre due to the presence of multiple synchronous ipsilateral tumours and proximity of one lesion to the nipple. She sought a second opinion at the authors' facility and was agreeable to a “trial of breast conservation treatment.” Localisation of the impalpable periareolar lesion and of the suspicious axillary lymph node was performed. She underwent an en bloc wide excision of the two left breast lesions through a boomerang incision and axillary staging through a separate axillary incision. The sentinel node coincided with the localised node and was found to be positive for metastasis on frozen section analysis. She underwent axillary dissection at the same operation. Histology was reported as a 4 cm invasive ductal carcinoma, with no intervening normal tissue between the clinical lesions. Three of sixteen axillary lymph nodes were involved. She is currently disease-free after more than 5 years.

**Figure 2:**
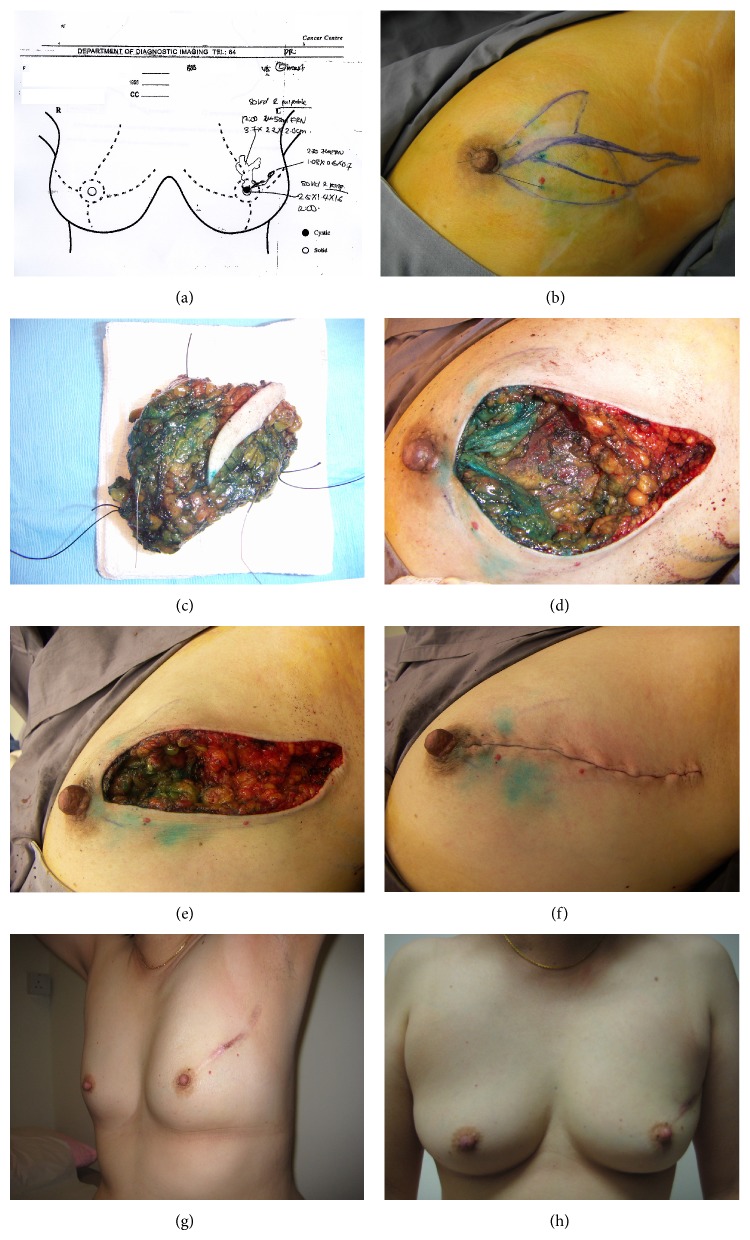
((a)–(h)) Avoiding mastectomy in a patient with “multicentric tumour” on imaging. This patient was diagnosed to have high grade ductal carcinoma in situ (DCIS) at an oncology centre and was offered mastectomy on the assumption that this was a multicentric lesion. She sought a second opinion with the authors and was agreeable to a “trial of breast conservation treatment.” Just prior to surgery, the lateral and medial extents of her dual-segment disease were localised under ultrasound guidance. Tissue resection was planned as indicated to balance the need for negative margins and retention of sufficient uninvolved parenchyma for defect repair. Through a radial incision and eccentric ellipse, an en bloc resection of the lesion using a multisegment resection pattern was performed. Sentinel node biopsy was performed through the same incision for this palpable high grade DCIS. Histology was reported as a unifocal 25 mm high grade DCIS. No multicentric component could be identified. She completed all adjuvant treatment and is now disease-free more than 5 years after surgery.

Although the presence of multicentric disease, or MFMCBC, is a conventional contraindication for BCT [36], a recent expert consensus considers this scenario a relative rather than an absolute contraindication [37]. MFMCBC pose technical challenges for BCT but may be surmounted with careful attention to surgical planning and technique. Patients initially considered ineligible for BCT by some surgeons might in fact be suitable candidates once appropriate measures are applied, avoiding “unnecessary mastectomies.” Examples of these situations are depicted in Figures 1 and 2. Based on imaging findings and percutaneous biopsy results, these two patients were recommended to have a mastectomy at another tertiary referral cancer centre. A second opinion was sought with the authors and they were willing to undergo a “trial of BCT.” Both underwent successful BCT and are now disease-free more than five years after surgical treatment. Sometimes, approaches which contravene conventional guideline recommendations are necessary. For example, unlike guidelines which recommend skin crease incisions [36], radial incisions may be necessary to incorporate larger lesions, multiple tumours, and allow adequate exposure for remodelling to avoid deformity [38, 39] (Figures 1 and 2).

Advances in imaging resulted in an improved ability to identify multiple tumour foci. However, recent data suggest that this increase in identification of MFMCBC with MRI results in higher mastectomy rates without definitive improvement in survival outcomes [40, 41]. Hence, a selective approach should be taken with the use of preoperative MRI. In this study, because preoperative MRI was not used, four patients, all of whom had multiple ipsilateral cancers, required a reoperation. MRI may be applied when multiple cancers or extensive microcalcifications are detected on conventional imaging to avoid reoperations. However, further work may be necessary in this area to assess specific selection criteria for its use to balance this need against a potential for increasing mastectomy use in this group of patients.

The reported rise in mastectomy rates is observed with a concomitant increase in the use of contralateral prophylactic mastectomy (CPM) [24, 25]. Factors contributing to this phenomenon include the use of preoperative MRI, the presence of multicentric disease, the availability of reconstructive surgery, fear of cancer recurrence, concerns over the risk of contralateral breast cancer (CBC), and a perceived survival benefit with mastectomy [24, 29]. The first three factors, which may also influence high mastectomy rates in Chinese populations, are clinician related and have been discussed earlier. The latter three involve patient psychology and information dissemination for decision-making. Data on how communication methods and decision aids affect patient's surgical decisions is varied [31–33, 42]. It is interesting to note that BCT tends to be preferred in women without breast cancer [43], but once diagnosed, higher mastectomy rates as the definitive operation were reported with greater patient involvement in decision-making [44]. In the light of recent data that higher mastectomy rates could lead to poorer survival outcomes, a reexamination of patient decision-making processes is needed. One factor which may influence patient choice in favour of mastectomy despite eligibility for BCT is the availability of reconstruction [24]. While reconstruction does lead to improved body image [26], its availability prior to a “trial of BCT” may have an adverse effect on BCT rates, possibly encouraging not only mastectomy but CPM as well. Some authors have concluded that, based on current evidence, the use of CPM for the purposes of risk reduction in sporadic breast cancer is unjustified [25, 44]. Other investigators share a different opinion [24]. Arriving at the delicate balance between a paternalistic approach recommending treatment associated with evidence-based superior outcomes and allowing patient autonomy in decision-making is complex and elusive. This difficulty can make a “simple can be harder than complex” situation in the pursuit of improvements in BCT [19]. Further work on this subject is warranted.

In a surgical era where there is increasing public acceptance towards less invasive procedures for equivalent outcomes in breast cancer, as has been seen in the paradigm shifts with percutaneous breast biopsies and the management of the axilla, it is perplexing that BCT rates are persistently low in predominantly Chinese communities, and mastectomy and CPM rates are rising in other populations. Recent data showing the potential for improved survival, lower complication rates, cost-effectiveness, and better body image during survivorship with BCT [6–10, 21, 25, 26] behoves clinicians to consider means of increasing its utilisation. The surgeon factor has been shown to affect BCT rates [32, 45, 46]. The data in this study appears to support this conclusion. It may be reasonable to surmise that surgeons recommending BCT apply operative techniques to surmount patient's physical attributes of SVB. Careful attention to clinical approaches and surgical technique may raise BCT rates in a predominantly Chinese community from approximately 30% to 86%, minimising unnecessary mastectomies in this population. Further work is needed to investigate if the concepts for improving utilisation of BCT in a population with prevailing low BCT rates may be applied in other settings to reverse a trend of rising mastectomy rates.

The retrospective nature of this study and small cohort are limitations to this study. In addition, the authors' practice in a private healthcare facility may serve as a selection bias where women who actively sought BCT were treated at the authors' facility. Notwithstanding, the distribution of tumour sizes is not dissimilar from other reports where oncoplastic reduction mammoplasty was performed [34, 35], indicating that the concepts discussed herein may be applicable to other healthcare settings to raise BCT rates.

## 5. Conclusion

Higher BCT rates demonstrated in this study than previously reported for predominantly Chinese communities suggest that it is possible to minimise unnecessary mastectomies in this select population. Further work is needed to define modifying factors.

## Figures and Tables

**Table 1:** Comparison of published data for BCT rates.

Author	Centre/country/study period	*n*	Characteristics	% BCT
Predominantly Chinese populations				
Sim et al. [11]	National Cancer Centre, Singapore (2001–2010)	5130	Stages 0–IV	29.2%
Wang et al. [12]	Changi General Hospital, Singapore (2002–2008)	761	Stages 0–IV	23.3%
Chang et al. [13]	National University Hospital, Singapore (1990–2007)	2449	Stages 0–IV	29.2%
Yip et al. [14]	University of Malaya Medical Centre (2001–2005)	953	T1, T2	29.7%
Yau et al. [15]	Pamela Y. Nethersole Eastern Hospital, Hong Kong (1994–2007)	2375	T1, T2	30%

International/Western				
Agarwal et al. [6]	SEER database (1998–2008)	132 149	Tumour ≤4 cm, ≤3 lymph node +	70%
McGuire et al. [16]	Moffitt Cancer Centre, FL, USA (1994–2007)	5865	Stages 0–IV	63.7%
Lee et al. [17]	University of Michigan Medical Centre, Michigan, USA (2003–2005)	993	Tis-T4	63%
Garcia-Etienne et al. [18]	EUSOMA (2003–2010)	15 369	Stages 0, I, and II (stage III, T3/T4 excluded)	73.3%
Current study	MammoCare, Singapore (2008–2011)	125	Symptomatic	82.4%
		46	Screen detected	95.6%
		171	Tis-T4	85.9%

BCT: breast conservation treatment.

SEER: surveillance, epidemiology, and end-result.

FL: Florida, USA: United States of America.

EUSOMA: European Society of Breast Cancer Specialists.

**Table 2:** Summary of demographic, clinicopathologic, and outcome data for study population.

Clinicopathologic characteristic	All patients (*n* = 171)		BCT (*n* = 147)		Mastectomy (*n* = 24)				*P* value
		(%)		(%)	By need (15)	(%)	By choice (9)	(%)	
Age in years									
Median (range)	48 (28–78)								
Mean (SD)	48.6 (10)		47.9 (10)		50.1 (8.8)				0.40
Mean (SD)			47.9 (10)				58.0 (6.9)		***0.003***
Ethnicity									0.88
Chinese	114	(66.7)	98/114	(86.0)	8/114	(7.0)	8/114	(7.0)	
Malay/Indonesian	12	(7.0)	10/12	(83.3)	2/12	(16.7)	0/12		
Indian	11	(6.4)	10/11	(91.0)	1/11	(9.0)	0/12		
Other Asian	14	(8.2)	13/14	(92.9)	1/14	(7.1)	0/14		
Caucasian	20	(11.7)	16/20	(80.0)	3/20	(15.0)	1/20	(5.0)	
Mode of presentation									0.07
Symptomatic tumours	125	(73.1)	103/125	(82.4)	13/125	(10.4)	9/125	(7.2)	
Screen detected lesions	46	(26.9)	44/46	(95.6)	2/46	(4.3)	0		
All patients	171		147/171	(85.9)	15/171	(8.8)	9/171	(5.3)	
Tumour size in mm (range)									
Median (range)	19.0 (3–97)		18.0 (3–72)		35.0 (4–97)		15.9 (3–35)		
Mean (SD)	21.1 (15.4)		19.2 (12.1)		40.5 (28.0)				***0.000***
(DCIS included)			19.2 (12.1)				18.3 (12.9)		0.83
≤20 mm	108	(63.2)	100/108	(92.6)	4/108	(3.7)	4/108	(3.7)	
21–50 mm	51	(29.8)	39/51	(76.5)	7/510020	(13.7)	4/51	(7.8)	
>50 mm	9	(5.3)	6/9	(66.7)	3/9	(33.3)	0		
T4	3	(1.8)	2/3	(66.7)	1/3	(33.3)			
Pathologic stage									***<0.001***
0	22	(12.9)	20/22	(90.1)	1/22	(4.5)	1/22	(4.5)	
I	70	(41.0)	69/70	(98.6)	1	(1.4)	0		
II	55	(32.2)	46/55	(83.6)	4/55	(7.3)	5/55	(9.1)	
III	21	(12.3)	11/221	(52.4)	8/21	(38.1)	2/21	(9.5)	
IV	1	(0.6)	0		1				
Unknown	2	(1.2)	1/2				1/2		
Histological type									0.34
DCIS	22	(12.9)	20/22	(91.0)	1/22	(4.5)	1/22	(4.5)	
Invasive ductal	132	(77.2)	114/132	(86.4)	11/132	(8.3)	7/125	(5.6)	
Invasive lobular	7	(4.1)	5/7	(71.4)	1/7	(1.4)	1/7	(1.4)	
Other invasive	10	(5.8)	8/10	(80.0)	2/10	(20)			
Grade									0.48
DCIS	22	(12.9)	20/22	(91.0)	1/22	(4.5)	1/22	(4.5)	
1	29	(17.0)	28/29	(96.5)	1/29	(3.6)	0		
2	61	(35.6)	50/61	(81.7)	6/61	(9.8)	5/61	(8.2)	
3	54	(31.6)	44/54	(81.5)	7/54	(13.0)	3/54	(5.6)	
Unknown	5	(2.9)	4/4	(100)					
Neoadjuvant medical therapy									***<0.001***
Yes	25	(14.6)	16/25	(64.0)	8/25	(32.0)	1/25	(4.0)	
No	146	(85.4)	131/146	(89.7)	7/146	(4.8)	8/146	(4.8)	
Disease extent									0.97
Unifocal	128	(74.6)	110/128	(85.9)	11/128	(8.6)	7/128	(5.5)
Multiple foci at diagnosis	43	(25.1)	34/40	(85)	4/40	(10)	2/40	(5)

BCT: breast conservation surgery; SD: standard deviation; DCIS: ductal carcinoma in situ.

**Table 3:** List of patients with posttreatment events.

Presentation	Treatment	Time to local recurrence	Time to distant recurrence	Treatment for recurrence	Comments/outcome
T3N1	Neoadjuvant chemotherapy, BCT, RT	Four months	Nil	Mastectomy	Disease-free at 57 months

T3N2	Neoadjuvant chemotherapy, mastectomy, RT	Nil	35 months, visceral, bony	Chemotherapy	Succumbed at 42 months

T2N2	BCT, adjuvant chemotherapy, RT	33 months	33 months, visceral	Declined treatment	Lost to follow-up

T3N3	Disease progression during neoadjuvant chemotherapy, mastectomy, RT	8 months	15 months, CNS	Declined further chemotherapy, VP shunt	Succumbed at 20 months

T3N1	Neoadjuvant chemotherapy, BCT, RT	Nil	9 months, CNS	Declined further chemotherapy	Died 13 months after surgery

T3N0	Neoadjuvant chemotherapy, BCT, RT	Nil	12 months, CNS	Chemotherapy	Died 19 months after surgery

BCT: breast conservation treatment.

RT: radiotherapy.

CNS: recurrence in the central nervous system.
